# Hemoglobin A_1c_ is associated with severity of coronary artery stenosis but not with long term clinical outcomes in diabetic and nondiabetic patients with acute myocardial infarction undergoing primary angioplasty

**DOI:** 10.1186/s12933-017-0578-7

**Published:** 2017-08-08

**Authors:** Jianqing She, Yangyang Deng, Yue Wu, Yulong Xia, Hongbing Li, Xiao Liang, Rui Shi, Zuyi Yuan

**Affiliations:** 1 Cardiovascular Department, First Affiliated Hospital of Medical College, Xi’an Jiaotong University, Xi’an, 710048 People’s Republic of China; 2 Key Laboratory of Environment and Genes Related to Diseases, Ministry of Education, Xi’an, 710048 People’s Republic of China; 3 Cardiovascular Department, First Affiliated Hospital of Beijing University, Beijing, 100005 People’s Republic of China

**Keywords:** HbA_1c_, Type 2 diabetes, Acute myocardial infarction, Coronary artery stenosis

## Abstract

**Background:**

Acute myocardial infarction (AMI) patients with type 2 diabetes mellitus are known to present with multiple vessel lesions during coronary angiography. The underlying mechanism remains elusive and there is a shortage of serum prediction markers. In this study, we investigate the relationship between admission HbA_1c_ and severity of coronary artery stenosis and subsequent prognosis in AMI patients with or without diabetes.

**Research design and methods:**

We measured admission HbA_1c_, and vessel scores based on the number of diseased coronary vessels with significant stenosis in 628 patients diagnosed with AMI. Simple and multi-regression analysis were performed to investigate the correlation between HbA_1c_ and the severity of coronary artery stenosis. Major adverse cardiovascular events (MACE), including new-onset myocardial infarction, acute heart failure and cardiac death, were documented during the follow-up. 272 non-DM participants and 137 DM participants were separated into two groups based on HbA_1c_ levels for survival analysis during a 2-year follow up.

**Results:**

448 non-DM patients and 180 DM patients were included in the initial observational analysis. 272 non-DM patients and 137 DM patients were included in the follow-up survival analysis. The admission HbA_1c_ level was found to be significantly positively correlated to the number of affected vessels suffering from significant coronary artery stenosis both in DM (R square = 0.012; 95% CI 0.002 to 0.623, P = 0.049) and non DM patients (R square = 0.025; 95% CI 0.009 to 0.289, P = 0.037). Kaplan–Meier survival analysis revealed no significant difference with regard to different HbA_1c_ levels either in DM or non-DM patients at the end of follow-up.

**Conclusions:**

In patients with AMI, admission HbA_1c_ is an important predictor for the severity of coronary artery stenosis in non-DM and DM patients. Further studies are needed to determine whether longer term follow-up could further identify the prognosis effect of HbA_1c_ on MACE.

## Introduction

Acute myocardial infarction (AMI) is one of the leading health threatening diseases in the world [1, 2] and it remains the most common cause for morbidity and mortality in patients with type 2 diabetes disease (T2DM) [3]. On the one hand, although AMI patients with T2DM are known to present with multiple vessel lesion during coronary angiography, the underlying mechanism remains elusive and there is a shortage of serum prediction markers [4, 5]. On the other hand, glucometabolic disturbance is common in AMI and is found to be associated with significantly increased rate of all-cause mortality, congestive heart failure and shock [6]. However, due to stress hyperglycemia, which commonly occurs secondary to increased catecholamine levels in AMI, plasma glucose level at the onset of AMI is considered not a good morbidity and mortality predictor [7, 8].

Glycated hemoglobin A_1c_ (HbA_1c_), measured primarily to identify 3-month average plasma glucose concentration, is accepted as a marker for long term glucose control in diabetes management [9]. Recent studies have revealed an association between chronic glucose dysregulation, assessed by HbA_1c_ levels, and prognosis of AMI [10]. However, although lowering HbA_1c_ levels is proven to have beneficial effects on microvascular complications [11, 12], the effects on macrovascular complications including AMI remain under explored.

It is reported that admission HbA_1c_ level is a prognostic factor associated with mortality after acute myocardial infarction [13]. Moreover, quantitative relationship between HbA_1c_ and atherosclerosis plaque textures is reported among diabetes mellitus (DM) patients with cardiovascular disease [14]. It is noteworthy that patients suffering from diabetes exhibit higher incidence of multi-vessel lesions during coronary artery angiography. However, it still remains unclear whether HbA_1c_ level correlates to the severity of coronary artery stenosis. Besides, although some studies identify HbA_1c_ as an effective marker in predicting major adverse cardiac events (MACE) in AMI patients with or without DM [15], others show no prognostic value or different results between diabetic and nondiabetic patients [16].

In this prospective cohort study, we investigate the relationship between admission HbA_1c_ and the severity of coronary artery stenosis in AMI patients with or without diabetes. We subsequently carry survival analysis to investigate the effects of admission HbA_1c_ levels on long term mortality and morbidity in AMI patients.

## Research design and methods

### Study design and participants

This was a single-center, prospective cohort study. Consecutive patients admitted to the cardiology department of the First Affiliated Hospital of Xi’an Jiaotong University for AMI between January 2013 and December 2016 were selected. The inclusion criteria were: (1) confirmed admission diagnosis of AMI, (2) successful treatment by angioplasty, (3) without diabetic ketosis or nonketotic hyperosmolar coma. The exclusion criteria were: (1) severe noncardiac disease with expected survival of less than 1 year and unwillingness to participation, (2) patients refusing angioplasty, (3) patients over the age of 80 years or living far away from the hospital’s catchment area. A patient could only be included once. Information about patients’ present medication, vascular risk factors and detailed medical history were obtained via questionnaires. Follow-up information was obtained via telephone questionnaires by the general practitioner (GP). AMI and DM were defined based on the universal definition criteria by the American Cardiology College and the American Diabetes Association criteria, respectively [17, 18]. Patients’ MACE, including new-onset myocardial infarction, acute heart failure and cardiac death, were documented during follow-up. Written informed consent was obtained from all study participants, with ethnic committee approval at the First Affiliated Hospital of Xi’an Jiaotong University.

### Assessment of HbA_1c_ and coronary artery stenosis

Blood HbA_1c_ levels of all patients were measured within 3 h of admission, regardless of whether they had been fasting, using Siemens DCA analyzer for quantitative assay of HbA_1c_ in blood. Both the concentrations of specific HbA_1c_ and the concentration of total hemoglobin were measured. The ratio was reported as percent HbA_1c_. The HbA_1c_ was studied as a continuous variable.

Selective coronary angiography was performed in multiple views. Coronary angiograms were analyzed by two experienced observers who were blinded to the identities and clinical information of the patients. Vessel scores were assessed based on the number of diseased coronary vessels with significant stenosis (greater than 50% stenosis of the lumen diameter). Next, the relationships between admission HbA_1c_ and the results of coronary angiography were statistically evaluated.

### Statistical analysis

All statistical analyses were performed by using SPSS for Windows 17.0 (SPSS Inc, Chicago, IL). Data were presented as frequencies and percentages for categorical variables and mean ± SD for continuous variables, unless otherwise indicated. One-way ANOVA was used to compare continuous variables. Simple linear analysis was used for calculating correlation between HbA_1c_ and the severity of coronary artery stenosis. To ascertain the independent contribution to coronary artery stenosis, multivariate regression analysis was conducted. Kaplan–Meier survival curve analysis was used to represent the proportional risk of all-cause mortality and MACE for the admission HbA_1c_ values in patients with or without DM. Patients were divided into two groups based on medians of HbA_1c_ levels in DM and non-DM groups separately. A value of P < 0.05 was considered statistically significant.

## Results

### Study population

**Fig. 1:**
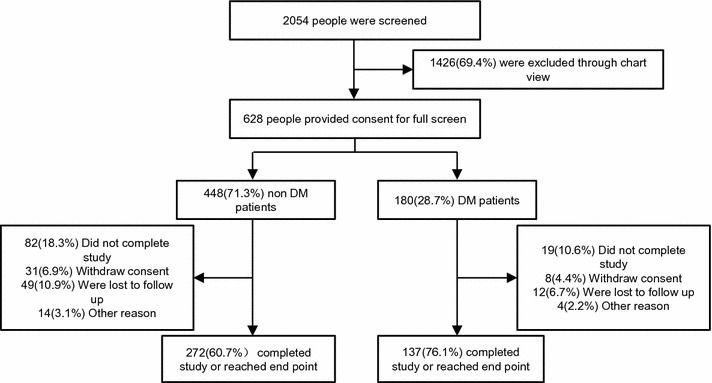
Enrollment an outcomes

From January 2013 till December 2016, a total of 2054 patients were enrolled in the study; 448 non-DM patients and 180 DM patients were included in the initial observational analysis, while 272 non-DM patients and 137 DM patients were included in the follow-up survival analysis (Fig. 1). The main reason for exclusion were that patients refused consent for full screen (53.2%), that they were over the age of 80 (16.5%), and that they refused angioplasty (5.9%). Baseline patients’ characteristics are shown in Table 1a for patients without DM and in Table 1b for patients with DM in whole and divided based on medians of HbA_1c_. The mean age was 59.84 ± 10.71 years in non-DM and 61.00 ± 10.41 years in DM patients. The mean HbA_1c_ was 5.73 ± 0.76% in non-DM and 7.86 ± 1.56% in DM patients. The median points of HbA_1c_ were 5.7% in non-DM and 7.1% in DM patients. No significant difference in risk factors at baseline were seen in different HbA_1c_ groups in either non-DM or DM patients.

**Table 1:** Basic characteristics for patients (a) without DM and (b) with DM

	Whole	HbA_1c_ (%)		P value
		≤5.7	>5.7	
(a)				
Patient number	272	142	130	
Average HbA_1c_ (%)	5.73 ± 0.76	5.46 ± 0.21	6.05 ± 0.18	<0.001
Age (years)	59.84 ± 10.71	59.79 ± 10.44	59.90 ± 11.03	0.93
Female (%)	20.96%	20.28%	26.15%	
BMI (kg/m^2^)	24.81 ± 3.15	24.96 ± 3.25	24.54 ± 3.02	0.4
Current/exsmoker (%)	57.72%	60.84%	56.77%	
Systolic BP (mmHg)	124.25 ± 20.07	124.49 ± 19.19	123.78 ± 17.83	0.75
DiastolicBP (mmHg)	77.44 ± 12.56	77.36 ± 11.26	77.45 ± 10.87	0.95
FBG (mg/dL)	6.05 ± 2.07	5.82 ± 1.61	6.15 ± 2.24	0.23
Ejection fraction (%)	59.25 ± 19.47	59.21 ± 13.01	59.12 ± 12.19	0.95
LDL-C (mmol/L)	2.28 ± 0.83	2.36 ± 0.72	2.19 ± 0.79	0.07
Creatine (µmol/L)	68.54 ± 15.72	68.75 ± 16.11	68.17 ± 15.19	0.76
Previous history of hypertension (%)	49.63%	51.05%	55.20%	
CHF (%)	6.99%	7.69%	6.27%	
Myocardial infarction (%)	16.18%	22.38%	24.74%	
PCI or CABG (%)	19.49%	18.88%	16.64%	
In hospital treatment aspirin (%)	95.96%	96.50%	95.67%	
β-Blocker (%)	82.72%	80.42%	78.66%	
Statin (%)	93.75%	97.20%	97.23%	
CCB (%)	30.88%	20.28%	15.44%	

	Whole	HbA_1c_ (%)		P value
		≤7.1	>7.1	
(b)				
Patient number	137	60	77	
Average HbA_1c_ (%)	7.86 ± 1.56	6.53 ± 0.36	6.53 ± 0.36	<0.001
Age (years)	61.00 ± 10.41	57.52 ± 10.15	64.05 ± 10.17	0.003
DM duration (years)	4.33 ± 5.91	2.83 ± 4.52	5.71 ± 6.67	<0.001
Female (%)	20.44%	16.70%	25.12%	
BMI (kg/m^2^)	25.80 ± 2.76	25.84 ± 2.57	25.78 ± 2.92	0.95
Current/exsmoker (%)	56.20%	73.29%	49.73%	
Systolic BP (mmHg)	127.99 ± 20.45	126.18 ± 22.40	129.40 ± 18.82	0.36
DiastolicBP (mmHg)	79.47 ± 11.80	79.61 ± 12.76	79.36 ± 11.07	0.9
Fasting plasma glucose	7.39 ± 2.95	7.22 ± 3.08	7.54 ± 2.82	0.47
Ejection fraction (%)	56.61 ± 18.07	56.73 ± 11.70	56.51 ± 11.84	0.92
LDL-C (mmol/L)	2.20 ± 0.93	2.35 ± 0.95	2.08 ± 0.84	0.08
Creatine (µmol/L)	71.68 ± 28.41	69.88 ± 21.91	73.08 ± 32.66	0.52
Previous history of hypertension (%)	59.85%	54.97%	55.83%	
CHF (%)	8.03%	6.69%	8.36%	
Myocardial infarction (%)	19.71%	20.02%	26.52%	
PCI or CABG (%)	24.82%	26.73%	35.80%	
In hospital treatment aspirin (%)	98.54%	40.28%	97.68%	
β-Blocker (%)	89.05%	96.64%	89.76%	
Statin (%)	99.27%	100.00%	100.00%	
CCB (%)	27.74%	21.68%	27.92%	
Insulin (%)	17.78%	13.64%	21.74%	
Metformin (%)	0.00%	0.00%	0.00%	
Acarbose (%)	35.56%	27.27%	43.48%	
Sulfonylureas (%)	13.33%	9.09%	17.39%	

Data are mean ± SD and number (%)

*DM* diabetes mellitus, *HbA*_*1c*_, hemoglobin A_1c_, *BMI* body mass index, *BP* blood pressure, *FBG* fasting blood glucose, *CHF* chronic heart failure, *PCI* percutaneous coronary intervention, *CABG* coronary artery bypass graft, *LDL-C* low density lipoprotein-cholesterol, *CCB* calcium channel blocker

### Association between HbA_1c_ and severity of coronary artery stenosis

**Fig. 2:**
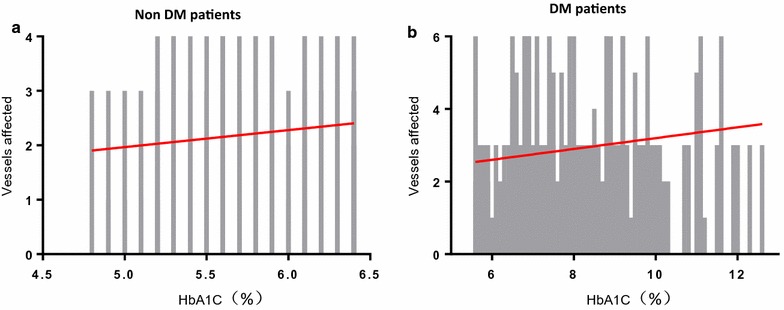
Simple linear analysis between HbA_1c_ and number of stenosis coronary arteries in non-DM and DM groups. **a** Simple linear regression model with HbA_1c_ level in relation to the number of stenosis coronary vessels in non-DM patients with AMI. **b** Simple linear regression model with HbA_1c_ level in relation to number of stenosis coronary vessels in DM patients with AMI

To investigate the relationship between HbA_1c_ and the severity of coronary artery stenosis, we utilized simple linear regression analysis. The admission HbA_1c_ levels were found to be significantly positively correlated with the numbers of affected vessels suffering from significant coronary artery stenosis both in DM (R square = 0.012; 95% CI 0.002 to 0.623, P = 0.049) and non-DM patients (R square = 0.025; 95% CI 0.009 to 0.289, P = 0.037) (Fig. 2; Table 2).

**Table 2:** Linear regression analysis of HbA_1c_ and coronary artery stenosis in patients with or without DM

Group	R square	Coefficient	95% CI	SEM	P value
Non DM	0.012	0.313	0.002 to 0.623	0.158	0.049
DM	0.025	0.149	0.009 to 0.289	0.071	0.037

*HbA*_*1c*_ hemoglobin A_1c_, *DM* diabetes mellitus, *CI* confidence interval, *SEM* standard error of measurement

Multi-regression analysis was then utilized to further determine the association of severity of coronary artery lesions and baseline characteristics including admission HbA_1c_. Interestingly, although HbA_1c_ level was found to be significantly positively correlated to the severity of coronary artery stenosis in DM patients (95% CI 0.040 to 0.312, P = 0.011), the P value was yet close to significance in non-DM patients (95% CI −0.648 to 0.032, P = 0.076) (Table 3a, b).

**Table 3:** Multi regression analysis of coronary artery stenosis in patients (a) without DM and (b) with DM

Factors	Coefficient	95% CI	SEM	P value
(a)				
Average HbA_1c_ (%)	−0.308	−0.648 to 0.032	0.173	0.076
Age (years)	0.003	−0.009 to 0.015	0.006	0.630
GRACE score	0.000	−0.003 to 0.003	0.002	0.744
HR (bpm)	0.001	−0.006 to 0.008	0.003	0.755
Systolic BP (mmHg)	0.003	−0.007 to 0.013	0.005	0.573
DiastolicBP (mmHg)	−0.008	−0.023 to 0.007	0.008	0.282
FBG (mg/dL)	0.024	−0.032 to 0.081	0.029	0.397
CKMB (U/L)	−0.001	−0.004 to 0.001	0.001	0.185
LDL-C (mmol/L)	0.092	−0.060 to 0.245	0.078	0.234
Creatine (µmol/L)	0.005	−0.003 to 0.012	0.004	0.225
(b)				
Average HbA_1c_ (%)	0.176	0.040 to 0.312	0.069	0.011
Age (years)	0.008	−0.013 to 0.030	0.011	0.454
DM duration (years)	−0.001	−0.037 to 0.035	0.018	0.954
GRACE score	0.004	−0.002 to 0.010	0.003	0.169
HR (bpm)	0.019	0.004 to 0.035	0.008	0.016
Systolic BP (mmHg)	0.000	−0.001 to 0.000	0.000	0.202
DiastolicBP (mmHg)	−0.011	−0.021 to −0.001	0.005	0.038
FBG (mg/dL)	0.183	0.115 to 0.251	0.034	0.000
CKMB (U/L)	−0.003	−0.007 to 0.001	0.002	0.160
LDL-C (mmol/L)	0.501	−0.465 to 1.468	0.489	0.307
Creatine (µmol/L)	0.027	−0.099 to 0.153	0.064	0.678

*HbA*_*1c*_ hemoglobin A_1c_, *DM* diabetes mellitus, *CI* confidence interval, *SEM* standard error of measurement, *GRACE* the global registry of acute coronary events, *HR* heart rate, *BP* blood pressure, *FBG* fasting blood glucose, *CKMB* MB isoenzyme of creatine kinase, *LDL-C* low density lipoprotein-cholesterol

### All cause mortality and MACE

At the end of the follow-up, within non-DM patients 14 (5.1%) patients died for all cause, 11 (4.0%) for cardiac cause, 30 (11.0%) for acute heart failure, and 12 (4.4%) had new-onset myocardial infarction. Within DM patients, 5 (3.6%) died for all cause, 2 (1.5%) for cardiac cause, 13 (9.5%) for acute heart failure, and 9 (6.5%) had new-onset myocardial infarction.

**Fig. 3:**
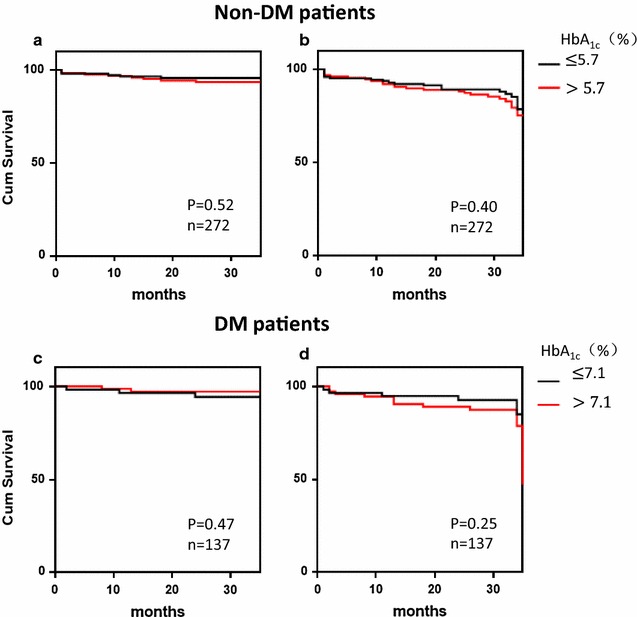
Kaplan–Meier survival curves for freedom from all cause mortality and MACE in non-DM and DM patient groups. **a** Kaplan–Meier survival curves for freedom from all cause mortality in non-DM by HbA_1c_ levels. P = 0.86. **b** Kaplan–Meier survival curves for freedom from MACE in non-DM by HbA_1c_ levels. P = 0.84. **c** Kaplan–Meier survival curves for freedom from all cause mortality in DM by HbA_1c_ levels. P = 0.62. **d** Kaplan–Meier survival curves for freedom from MACE in DM by HbA_1c_ levels. P = 0.34. There is not significant higher event-free survival rate in high HbA_1c_ level patients in two groups

Kaplan–Meier survival analysis was utilized to evaluate the survival curve in different HbA_1c_ groups in non-DM and DM patients. The analysis revealed no significant difference with regard to different HbA_1c_ levels both in DM and non-DM patients, shown in Fig. 3.

## Discussion

In this study, serum HbA_1c_ is found to be associated with the severity of coronary artery stenosis in diabetic and nondiabetic patients with acute myocardial infarction undergoing primary angioplasty. Moreover, admission serum HbA_1c_ level exhibits no effect on all cause mortality rate and MACE rate either in non-DM or DM patients in 2-year follow-up.

The important implication of the present study is that HbA_1c_ is identified as a serum predictor for severity of cardiovascular lesions in non-DM and DM patients. Previous study indicates a non-linear relationship between HbA_1c_ and major vascular outcomes and mortality [16]. It is also known that poor glycemic control is associated with multiple vessel lesions in coronary artery disease [19, 20]. However, few studies have addressed the question whether HbA_1c_ is related to the severity of coronary artery stenosis under non-DM and DM background. The major outcome of this study shows a positive correlation between admission HbA_1c_ concentrations and the number of stenosis coronary arteries in patients with acute myocardial infarction, indicating that HbA_1c_ level is a potential indicator for multiple coronary vessel lesions. Moreover, the significant correlation between HbA_1c_ and coronary artery stenosis is identified both in non-DM and DM population, implying that HbA_1c_ level monitoring might be beneficial even for patients without diabetes.

Acute hyperglycemia has been reported to be associated with acute adrenergic signal of stress [16, 21] and endothelial cell dysfunction in acute myocardial infarction, which is partially attributed to endothelial cell apoptosis [22, 23], reactive oxygen species (ROS) over production [24], and inflammation [25]. However, the effect of chronic hyperglycemia on vascular function and coronary artery complications has been less reported. It is shown before that long-term glucose dysregulation is associated with increased cell death signaling [26], inflammatory changes and fibrosis [27], and subsequent cardiomyopathy [28]. In this study, long term uncontrolled glucose level, indicated by HbA_1c_ is shown to be correlated with the severity of coronary artery complications. Besides, this correlation could be further identified in patients without diabetes but with relatively higher level of HbA_1c_.

There is conflicting evidence regarding HbA_1c_ level and major cardiovascular outcomes [13, 16, 29, 30]. In this study, HbA_1c_ is not associated with all cause mortality and MACE in both non-DM and DM group over a 2-year follow up. The less clear association between HbA_1c_ and cardiovascular outcomes could be due to a limited number of patients with a relatively short follow-up in the present study [16]. It is speculated that HbA_1c_ may have limited predictive power for short-term prognosis in patients with AMI, but its association with long-term prognosis may be stronger [31]. As a result, more well-designed and long-term studies as well as systemic analysis are needed to investigate whether HbA_1c_ will play an important role in the prognosis of AMI.

## Study limitations

This is a single centre based observational cohort study. The sample size in this study is relatively small, especially in patients with T2DM, therefore, comparisons of some subgroups may lack power to detect significant differences for selected variables. Although HbA_1c_ level is associated with the number of the stenosis coronary arteries, a complex and systemic score, i.e. SYNTAX score, could be further recorded to predict the severity of coronary artery stenosis more accurately.

## Conclusions

In patients with AMI, admission HbA_1c_ may be an important predictor for severity of coronary artery stenosis in non-DM and DM patients. The results of this study further support the view that chronic glycemic control should be one of the treatment targets for AMI patients. Further studies are needed to determine whether longer term follow-up would further identify the prognosis effect of HbA_1c_ on MACE.

## Abbreviations

HbA_1c_: hemoglobin A_1c_
AMI: acute myocardial infarction
T2DM: type 2 diabetes mellitus
MACE: major adverse cardiovascular events
GP: general practitioner
BMI: body mass index
BP: blood pressure
HR: heart rate
FBG: fasting blood glucose
CKMB: MB isoenzyme of creatine kinase
LDL-C: low density lipoprotein-cholesterol
CHF: chronic heart failure
PCI: percutaneous coronary intervention
CABG: coronary artery bypass graft
CCB: calcium channel blocker
GRACE: the global registry of acute coronary events
